# Satisfaction and Acceptability of Telemonitored Home-Based Exercise in Patients With Intermittent Claudication: Pragmatic Observational Pilot Study

**DOI:** 10.2196/18739

**Published:** 2021-03-22

**Authors:** Nils Cornelis, Roselien Buys, Tijl Dewit, Dries Benoit, Jomme Claes, Inge Fourneau, Véronique Cornelissen

**Affiliations:** 1 Department of Rehabilitation Sciences Group Rehabilitation for Internal Disorders KU Leuven Leuven Belgium; 2 Department of Cardiovascular Sciences Vascular Surgery KU Leuven Leuven Belgium

**Keywords:** eHealth, telerehabilitation, intermittent claudication, pilot

## Abstract

**Background:**

Current guidelines recommend supervised exercise training (SET) as a first-line treatment in patients with intermittent claudication (IC). SET has been shown to be more effective than home-based exercise therapy (HBET). However, the lack of available SET programs hampers broad SET implementation in clinical practice.

**Objective:**

The aim of this study is to assess patient satisfaction and acceptability of a structured HBET program using wearable technology and elastic band resistance exercises.

**Methods:**

A total of 20 patients with IC (Rutherford 1-3) with internet access and currently not engaged in structured exercise training were recruited in a pragmatic observational pilot study. Participants were instructed to complete 3 walking sessions and 2 elastic band resistance exercise sessions per week in their home environment during a 4-week period. Patient satisfaction and acceptability were assessed using a 5-point Likert scale questionnaire (1-2=very unsatisfied, 3=neutral, and 4-5=very satisfied) evaluating the materials and intervention content. Secondary outcomes were evaluated at baseline and at completion of the 4-week intervention and included maximal walking distance (MWD) and pain-free walking distance (PFWD), physical fitness, and patient-reported outcomes on quality of life, walking capacity, levels of kinesiophobia, and self-efficacy. Statistically significant changes were tested using paired *t* tests or Wilcoxon signed-rank tests.

**Results:**

All patients (15 men, 5 women; mean age 64.6, SD 10.6 years; range 41-81 years) completed the 4-week intervention and were highly satisfied with the program (mean overall score 4.5, SD 0.5). Patients’ questionnaire responses documented willingness to recommend the exercise program to other patients (mean 4.5, SD 0.5; median 4.5) and preference for continuing the intervention (mean 4.3, SD 0.5; median 4). Furthermore, participants endorsed the use of the sports watches to track walking sessions (mean 4.25, SD 0.6; median 4), felt safe (mean 4.4, SD 0.6; median 4), and appreciated personal feedback (mean 4.55, SD 0.5; median 5) and flexibility of training (mean 4.1, SD 0.7; median 4). Resistance training was not preferred over walking training (mean 2.65, SD 0.8; median 3). In addition, PFWD (+89 m; *P*=.001), MWD (+58 m; *P*=.03), Walking Impairment Questionnaire distance score (+0.18; *P*=.01), activity-related scores (+0.54; *P*<.001), and total quality of life (+0.36; *P*=.009) improved following the intervention. Other patient-related outcomes, physical fitness, and physical activity remained to be statistically unaltered.

**Conclusions:**

Patients with IC were satisfied and accepted technology to monitor and guide HBET, with observed short-term effectiveness regarding walking capacity and quality of life. However, elastic band resistance exercises as a part of HBET were not preferred over progressive walking.

**Trial Registration:**

ClinicalTrials.gov NCT04043546; https://clinicaltrials.gov/ct2/show/NCT04043546

## Introduction

### Background

Lower extremity artery disease (LEAD) is a chronic disease characterized by progressive atherosclerotic narrowing of the lower limb arteries. As such, insufficient blood flow to active muscles during exercise may result in complaints of intermittent claudication (IC), which often presents as cramping or burning-like pain during physical activities. Although not immediately life-threatening, LEAD and IC have a great impact on patients’ functional status and quality of life [1] through long-term pathophysiological changes (eg, atrophy, muscle weakness, reduced cardiorespiratory fitness) [2-4]. Furthermore, IC limits the ability to be physically active, enhancing the risk of serious cerebral and cardiovascular events [5].

Recent guidelines emphasize the importance of a first-line lifestyle-oriented approach when consulting with IC [6]. In this context, supervised exercise and walking in particular are cornerstone therapies that result in clinically significant improvements in pain-free walking distance (PFWD) and maximal walking distance (MWD) [7]. Meta-analytic research has shown that direct supervision of exercise training (SET) is a major contributor to progression in walking capacity [8]. However, SET is not readily available in most European countries, with only 30% of vascular surgeons having direct access [9,10]. Furthermore, even when SET is available, patients’ participation is low, mainly because of a lack of transportation and time [11,12]. In addition, reimbursement issues and lack of uptake in health policy plans further hamper the widespread use of SET [13]. As a result, next to optimal pharmacological treatment, first-line IC management is often limited to a less-effective Go-Home-And-Walk advice. Structured home-based exercise therapy (HBET) seems promising to bridge the gap between Go-Home-And-Walk advice and the underuse of SET programs [9,10]. Although recommended as the best available therapy when SET is unavailable [6], evidence supporting HBET programs is considerably scarce [7,14]. However, it is noteworthy that the first HBET studies included only general advice to exercise, relied on patient recall, and did not incorporate behavioral change techniques [15,16]. A more recent meta-analysis by Golledge et al [17] showed that when HBET was more structured (and monitored), the effectiveness of HBET in improving walking performance and physical activity was increased. Furthermore, the importance of regular contacts empowering behavioral change and a therapeutic relationship is crucial for success [16,18,19].

At present, eHealth technologies offer valuable tools to elicit the full potential of HBET [20]. Currently, eHealth, referred to as *telerehabilitation* in cardiac rehabilitation, includes exercise supervision (*telemonitoring*), guidance of exercise (*telecoaching*), and promotion of a healthy lifestyle [21]. Telerehabilitation interventions, such as telephone or internet-based coaching, designed to increase physical activity behavior and compliance to exercise therapy, have already proven to be feasible and effective in cardiac patients [22,23]. Moreover, recent advancements in commercial wearables offer a unique opportunity to monitor physical activity and exercise and support behavioral changes toward an active lifestyle [24]. As such, wearable technology might help to bridge the gap by preserving the patient-provider relationship and offering home-based structured exercise therapy of adequate intensity in a health care system under pressure [14].

However, one needs to address the needs and interests of all stakeholders, including patients [21]. In this line, a previous cohort study from our group showed that 81% of patients owning a computer and telephone were interested in telecoaching [25]. In addition, most patients preferred home-based exercise [26], and physiotherapists showed utmost interest (89%) in GPS tracking to monitor these sessions [27]. With regard to the mode of exercise, most guidelines highlight the use of walking intervals until experiencing moderate-to-severe IC pain to improve walking distance [16]. However, resistance training is also considered to be an effective exercise mode and offers the potential to induce a pain-free exercise stimulus [28]. Furthermore, in addition to offering general health-related benefits, the addition of resistance exercises seems promising in terms of disease-specific measures [29] in patients with IC. However, the most recent review did not include any home-based resistance training alternatives, although elastic band exercises might be an effective home-based solution [28].

### Objectives

In this exploratory, pragmatic observational pilot study, we primarily aimed to evaluate patient satisfaction and acceptability of a structured model of HBET using wearable technology during walking, in combination with home-based resistance exercises. In addition, we aimed to report on the adherence and potential effectiveness of this combined intervention on walking capacity, physical fitness, physical activity levels, and quality of life in the development of an HBET program for patients with IC.

## Methods

### Study Design

We conducted a 4-week exploratory observational cohort study to assess patient satisfaction and acceptability of an experimental HBET program combining walking therapy with elastic band exercises. The study was approved by the Ethical Committee of UZ (ethics approval number: S59686; Belgian registration: B322201630074) Leuven/KU Leuven (Leuven, Belgium) and registered on ClinicalTrials.gov (NCT04043546).

### Participants

Patients consulting the ambulatory vascular center of the University Hospitals Leuven (Leuven) between October 2017 and July 2018 were recruited by vascular surgeons. Using convenience sampling, we aimed to recruit 20 patients. Eligibility criteria included patients presenting with LEAD (Ankle-Brachial Index [ABI] ≤0.9 and/or a 15% decrease in ABI after a maximal treadmill test) and new-onset or conservatively treated IC (Rutherford I-III). Patients were excluded if they (1) had already participated in a structured, regular exercise program (eg, weekly physiotherapy); (2) showed exercise-induced signs of myocardial ischemia or complex ventricular arrhythmias during maximal treadmill exercise; (3) did not receive medical clearance for exercise; or (4) did not have access to a computer or the internet.

### Intervention

**Figure 1:**
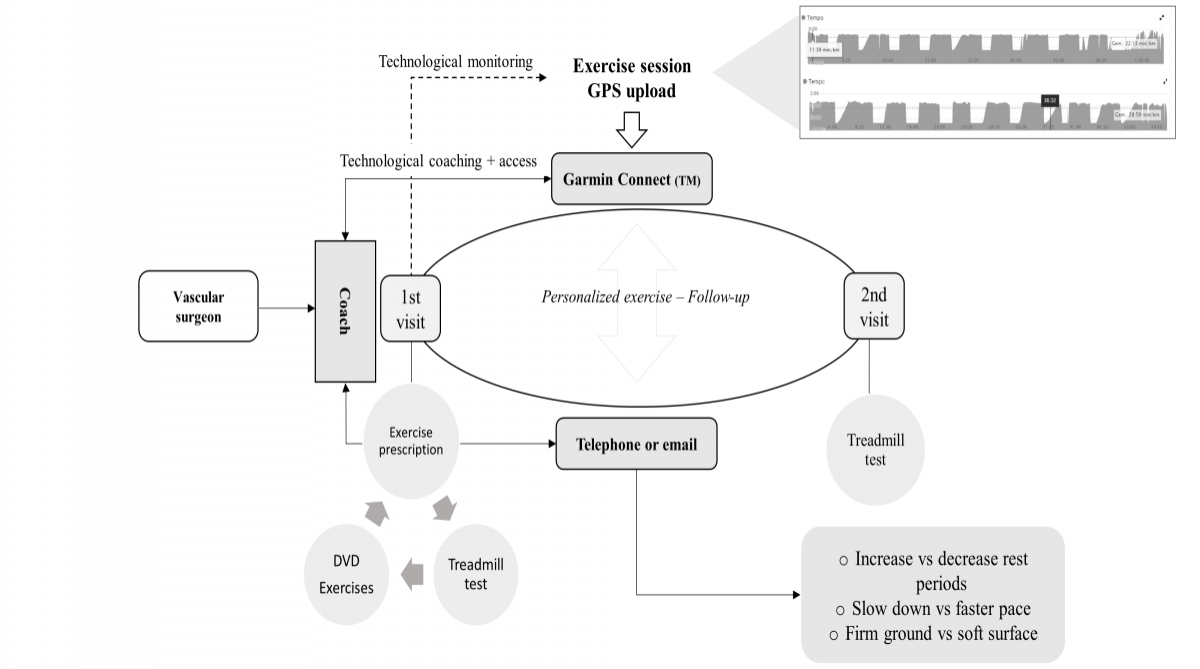
Pilot 4-week exercise intervention flow: baseline testing was done to provide a personal exercise program. The exercise program was monitored through GPS-derived data, uploaded by the participant. Telecoaching was provided through telephone or email.

The flow of this study is schematically depicted in Figure 1. To guide the 4-week home-based exercise program, participants were offered an informative booklet, a self-developed DVD with demonstration of the resistance band exercises (Multimedia Appendix 1), and a Garmin Forerunner 210. The booklet provided background information about the symptoms of IC, a person-tailored walking prescription with a logbook, and images to illustrate the resistance exercises.

### Walking and Resistance Program

The exercise intervention consisted of 3 walking days and 2 resistance training days each week. Walking was prescribed according to the Dutch activity guidelines for IC [30] and person tailored (eg, adjustment of walking speed, hilly terrain, duration of rest period, unsteady surface) to elicit only moderate claudication pain during 2- to 10-minute intervals. Interval breaks were generally 1.5 to 2 minutes depending on pain recovery. Walking sessions were monitored and evaluated using GPS-derived data from the web-based Garmin Connect platform. Resistance training consisted of 4 elastic band exercises: plantar flexion, hip flexion, hip extension, and hip abduction. The appropriate resistance was selected during a single familiarization session at baseline to successfully complete the prescribed 2 sets of 12 repetitions for each leg. According to their individual preferences, participants received feedback twice weekly to only once during the 4-week intervention period via telephone or email. Exercise therapy was monitored and guided by a physiotherapist (NC) who progressively adjusted the volume and intensity over the 4-week period. This was personalized during contact moments using subjective reflection from the patients, baseline treadmill tests, and GPS-derived data. As such, participants had the possibility to self-monitor their walking sessions, received timely feedback on their performance, and were provided with information on how to adapt their walking program [31].

### Outcome Measures

After a consultation at the vascular center, participants were invited for baseline and 4-week follow-up measurements at our research laboratory (University Hospitals Leuven), as shown in Figure 1. Doppler measurements from the latest clinical evaluation at the ambulatory vascular center were used to report the ABI of the most affected leg. Similarly, sociodemographic and clinical characteristics (eg, Rutherford classification) were derived from the electronic patient records of the last clinical consultation. In addition, the feasibility of physical activity assessment was evaluated at baseline and after 3 months.

#### Primary Outcome Measures: Patient Satisfaction and Acceptability

Patient satisfaction and acceptability of HBET were evaluated using a feedback survey adapted from Learmonth et al [32]. Patients were asked to rate the HBET, offered materials, coaching, and exercise prescription on a 5-point Likert scale (ie, *very unsatisfied, unsatisfied, neutral, satisfied, and very satisfied*). In addition, the participants were asked to provide written feedback on the received intervention and to provide suggestions for improvement.

Furthermore, all communication logs (telephone calls and emails) were registered and adherence to exercise was assessed using walking uploads and self-reported walking or resistance logs provided by the participant. Adherence was defined as the ratio of the number of exercise sessions to the number of prescribed exercise sessions.

#### Secondary Outcomes

##### Walking Capacity

Participants performed a progressive treadmill test using the Gardner protocol [33]. The walking speed was set at 3.2 km/h and adjusted (SD ±1 km/h) if needed. Every 2 minutes, we increased the inclination by 2% to a maximum of 10%. Participants were asked to report the onset and maximally tolerated claudication pain. Patients without IC symptoms who limited their walking capacity on the treadmill were excluded from this analysis. In addition, we used the Walking Impairment Questionnaire (WIQ) [34] to evaluate the walking distance, walking speed, and stair climbing capacity, with lower scores indicating greater impairment.

##### Quality of Life, Exercise Self-Efficacy, and Kinesiophobia

Patients were asked to fill in VascuQoL, a disease-specific questionnaire to assess quality of life. VascuQoL contains 25 seven-point Likert statements to measure the activity, symptom burden, pain, emotions, and social consequences related to LEAD [35]. Total scores and subscores for the VascuQoL questionnaire ranged from 1 to 7, with higher scores indicating a better quality of life. In addition, the Exercise Self-Efficacy Scale (ESES) was used to evaluate participants’ confidence in overcoming personal and environmental barriers to be physically active [36]. The ESES has a total score of 40 (highest level of exercise self-efficacy), combining 10 statements scored with a 4-point ordinal outcome. Finally, kinesiophobia, or movement-related fear of pain, was evaluated using the Tampa Scale of Kinesiophobia (TSK) [37], which assists in identifying participants avoiding physical activity because of unjustified pain beliefs. The TSK is scored on a 17-item questionnaire, with higher scores (4-point Likert scale) indicating elevated levels of kinesiophobia. A cut-off score of ≥37 was used to diagnose kinesiophobia [37].

##### Physical Fitness

Physical performance was assessed using the Short Physical Performance Battery (SPPB) and the Timed-Up-and-Go (TUG) test, with patients wearing their shoes. The SPPB evaluates the standing balance, 4-m gait speed, and lower extremity strength [38]. Each category of SPPB is scored from 0 to 4, resulting in a maximum score of 12 points, with higher scores indicating better performance. The TUG test is a functional test that evaluates functional chair stand and walking flexibility [39]. Participants were instructed to stand up from a chair, walk fluently around a 3-m separated cone, and sit down again. We used the fastest time for the 2 attempts in the analysis.

##### Physical Activity

Participants were instructed to wear a Sensewear (R) Mini device (Bodymedia) on the right upper arm for 7 days to measure the daily physical activity levels. An assessment was considered valid if the patient had worn it for at least 3 weeks and 2 weekend days with 90% daily (24-hour measurement) wear time [40]. Physical activity intensity was categorized as light (1.5-2.9 metabolic equivalents [METs]), moderate (3.0-5.9 METs), and (very) vigorous (≥6 METs). Sedentary behavior included all activities below a threshold of 1.5 METs. In addition, steps were registered to assess walking activities in daily life.

### Statistics

All data were presented as median and IQR or mean and SD. Normality of data was evaluated using the Shapiro-Wilk test. Statistical analyses were performed using JASP 0.11.1 (University of Amsterdam), with pre-post parametric (paired two-tailed *t* test) and nonparametric equivalent (Wilcoxon signed-rank) tests. An alpha level of 5% (two-sided) was used for statistical significance. No power calculations were performed on the study outcomes.

## Results

### Participants and Data Collection

**Figure 2:**
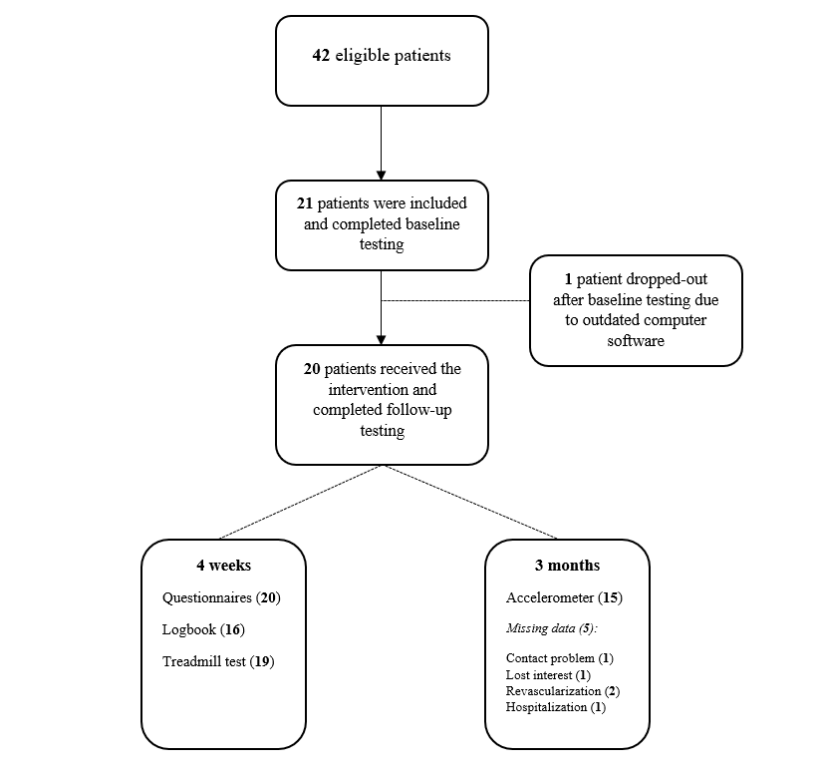
Flowchart with study inclusion and final analysis.

Out of 41 eligible patients, 21 (50% recruitment success) volunteered to participate (15 men and 6 women). A total of 3 patients were referred for additional cardiologic screening after baseline measurements because of presumed cardiac ischemia, complaints, or arrhythmias. Consequently, for 1 participant (P1), the intervention start was postponed, resulting in a 75-day interval period between measurements. One patient was excluded after recruitment. Our participants’ average age was 64.6 years (SD 10.6; range 41-81 years) and heterogeneous with regard to comorbidities, walking capacity, claudication location, duration of symptoms, and severity of disease (ABI; mean 0.65, SD 0.20; Rutherford classification [3 in 50%]). Moreover, all participants had dyslipidemia, 70% were hypertensive, 25% had diabetes, and 85% were ex-smokers or were still smoking. Individual demographic characteristics are detailed in Multimedia Appendix 2, and the study flow is presented in Figure 2. Baseline and follow-up measurements were completed within a median time period of 36 days (IQR 6), which corresponds to a median intervention time of 32 days (IQR 5).

### Primary Outcomes

**Figure 3:**
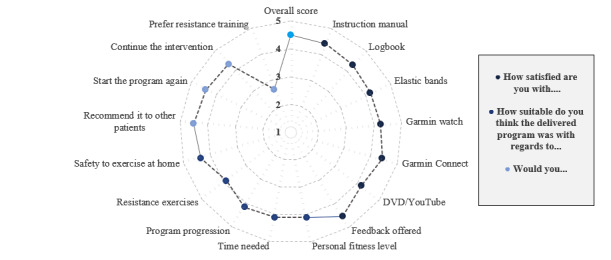
Feasibility of the intervention as scored by a 5-point Likert scale (mean scores). Range of scores: 1 (very dissatisfied or unsuitable), 2 (dissatisfied or unsuitable), 3 (neutral), 4 (satisfied or suitable), and 5 (very satisfied or suitable). Missing values: instruction manual (1), logbook (3), Garmin Connect (1), DVD or YouTube-link (3), personal fitness level (2), time needed (1), program progression (1), resistance exercises (1), safety to exercise at home (1), starting the program again (1), and continuing the intervention (1).

All users were very satisfied (mean overall score 4.5, SD 0.5; median 4.5, range 4-5) with the HBET program. These results were reflected in high adherence to the prescribed walking sessions (GPS and logbook combined=mean 89%, SD 25; GPS only=mean 86%, SD 28), with 75% (15/20) of the patients completing all prescribed walking sessions. In contrast, patients were less compliant with resistance training (mean 85%, SD 22; 56% (9/16) completed all prescribed sessions and 20% (4/20) of patients did not return their logbook) and did not prefer this exercise alternative over conventional walking therapy (mean 2.65, SD 0.8; median 3, range: 1-5). Intervention satisfaction scores regarding materials, feedback, personalization, and content of the intervention are depicted in Figure 3. Furthermore, it is important to note that participants perceived the home-based program as safe (mean 4.4, SD 0.6; median 4, range 3-5). Most participants also stated that they would re-enroll in the exercise program (mean 4.4, SD 0.5; median 4, range 4-5) and would recommend it to their peers (mean 4.5, SD 0.5; median 4.5, range 4-5). Qualitative reporting revealed that participants were positive about the option to visualize progression using the recorded training logs (n=2) or trigger to improve (n=2), personal guidance (n=2), and flexibility (n=2). However, resistance training (n=7) and pain during sessions (n=2) were perceived as less enjoyable.

In addition, we registered the number of telephone and/or email contacts. A median of 5 contacts during the 4-week intervention was provided for each patient: 3 follow-up contacts, 1 contact moment to provide technical assistance, and 1 contact combining the aforementioned. In addition, most contacts were provided through email (median 3) as compared with telephone calls (median 2).

### Secondary Outcomes

#### Walking Capacity

At baseline, MWD ranged between 141 and 828 m (median 414 m, IQR 253 m), with 2 patients being stopped by the investigator as claudication symptoms were not limiting the exercise test. In addition, 1 patient (*P2*) experienced claudication symptoms but stopped both tests because of gastric problems. Patients (n=3) were excluded from MWD analysis. Participants improved their PFWD and MWD compared with baseline, with a mean progression of +89 (SD 95) and +58 m (SD 97), respectively (*P*<.001 and *P*=.03; Multimedia Appendix 2). Similarly, the WIQ distance score (+0.18; *P*=.01) was significantly higher after the intervention. As no statistically significant change was established in WIQ speed (+0.03; *P*=.53) and WIQ stair climbing score (+0.02; *P*=.55), the overall WIQ score remained to be statistically unaltered (+0.08; *P*=.06; Multimedia Appendix 2).

#### Quality of Life, Exercise Self-Efficacy, and Kinesiophobia

Quality of life was better after the intervention (+0.36 on total VascuQoL; *P*=.009). The main areas of improvement were pain (+0.41; *P*=.04), physical activity (+0.54; *P*<.001), and emotions (+0.33; *P*=.06). No changes were noted in the social (+0.08; *P*=.56) and symptom (+0.15; *P*=.30) subscores. Kinesiophobia was elevated at baseline, with a median score of 38 (IQR 8.50). Self-efficacy (ESES) and kinesiophobia did not change (*P*=.18 and *P*=.17, respectively; Multimedia Appendix 2).

#### Physical Activity and Physical Fitness

At baseline, physical activity values for valid days were averaged for each participant, resulting in a median of 59 minutes of moderate to vigorous physical activity (IQR 63 minutes) per day. Moderate physical activity was the main contributor to daily physical activity in our sample, as 80% (16/20) of our sample did not reach 5 minutes of vigorous physical activity (median 2 minutes, IQR 4.3 minutes). In addition, our participants completed a median of 5297 (IQR 3118) steps per day. Follow-up data did not show any significant changes after 3 months. With regard to physical activity data acquisition, 95% (19/20) of participants fulfilled the targeted 90% daily Sensewear on-body time for at least 3 weeks and 2 weekend days at baseline. In contrast, 25% (5/20) of the patients did not complete the physical activity assessment after 3 months. Furthermore, the 4 follow-up measurements did not fulfill our strict validity criteria. Consequently, only 55% (11/20) of the participants had follow-up physical activity data. More information is provided in Multimedia Appendix 2, with elaboration on the encountered methodological issues. Physical performance (SPPB total score) was not significantly different (*P*=.06) after the intervention (Multimedia Appendix 2).

## Discussion

### Principal Findings

This study evaluated the satisfaction, acceptability, adherence, and potential effectiveness of a novel home-based exercise intervention that combines resistance training and walking therapy using wearables to monitor and guide patients with IC. Although our sample of 20 conservatively treated patients was heterogeneous in nature, participants generally perceived the exercise program with personalized feedback and monitoring as (very) positive. However, contrary to our hypothesis, elastic band exercises were not preferred over traditional walking sessions. Furthermore, we also found beneficial effects on quality of life (VascuQoL), subjective walking distance (WIQ), and objective walking distances (PFWD and MWD). Despite the short intervention duration, a clinically relevant improvement was found in the WIQ distance score [41]. As this study was designed to primarily evaluate patients’ satisfaction and acceptance, our results complement contemporary pilot studies in the field of eHealth solutions in patients with IC [20,42-45]**.**

We used commercially available wearables supported by GPS tracking to guide and monitor walking training. The exercise uploads showed additional value to evaluate adherence and guide personalized exercise prescription in our study. Researchers have already explored the advantages of GPS-derived walking information to evaluate community-based walking in patients with IC [46,47]. They found an acceptable 0.81 correlation comparing free-living PFWD and results from a standardized treadmill test documenting its usefulness for the evaluation of walking distances [46]. As such, wearables offer possibilities to assess physical activity levels and monitor [48], guide, and evaluate progress in future structured home-based exercise programs [46] (Figure 1). Recently, Dusha et al [44] reported on their 12-week pilot study in 10 patients in which they used commercial step counters with adapted coaching that resulted in improved walking capacity in patients with IC. Conversely, the largest trial to date—Home-Based Monitored Exercise for the PAD (HONOR) study [43]—did not provide feedback based on the uploaded exercise information. Patients only received monthly feedback for the last 4.5 months during the 9-month HONOR intervention, which might explain the unchanged walking frequency compared with usual care after 9 months. Our participants asked for and received weekly feedback. Therefore, incongruity between the use of activity trackers to increase the overall physical activity (eg, daily steps) and specific exercise recommendations with appropriate, direct feedback might explain the lack of improvement [43]. In summary, the appropriate use of technology seems mandatory to provide a symbiosis between the wearable (*tool*) and the intervention (*goal*), which is generally acceptable to patients with IC.

The novelty of this study was the incorporation of home-based resistance training. Although more than 80% stated that they were interested in using elastic bands as an alternative to walking therapy [25], patients now rated the addition of elastic band exercises as neutral or negative compared with walking. This was somewhat surprising, as pain is the most cited barrier to exercise [25]**,** and resistance exercises were anticipated to result in less pain in terms of oxygen demand in the lower legs [28]. However, similar results were noted in geriatric inpatients, where objective measures of elastic band use contrasted with positive attitudes of staff and patients regarding the benefits [49]. Although no specific reasons were provided, we hypothesize that highly prevalent musculoskeletal comorbidities in patients with IC (eg, lumbar spine disease in 75.7% [50]) and lack of direct supervision might have hampered the correct execution of the elastic band exercises. Quality of execution has been proposed as an important driver of improved adaptation after supervised resistance programs compared with nonsupervised programs in older adults [51]. Therefore, direct supervision appears to be essential when prescribing technically challenging exercises.

Moreover, it is interesting to note that 60% (12/20) of our sample experienced some degree of kinesiophobia (ie, TSK≥37) at baseline. Compared with the significant changes observed in terms of walking outcomes, no change occurred at the level of fear avoidance. This discrepancy might be evoked by the short intervention period or the lack of patient education to *explain the pain* and induce behavioral change. These findings once again emphasize the importance of addressing these beliefs when designing an exercise intervention, as they might interfere with exercise therapy perception and adoption [52,53]. In addition, the importance of the patient-provider alliance using in-person visits may not be overlooked when designing telemonitored exercise programs [18,43]. Therefore, the development of so-called hybrid interventions [44] might bridge this gap, which has been shown in an earlier successful trial using step monitors [54]. Therefore, future studies should investigate the add-on effect of direct supervision in home-based interventions to (1) evaluate patient perception and methods to implement resistance exercises and (2) reduce activity-related fear using behavioral change or educational interventions.

Furthermore, this study also included a feasibility evaluation of the different assessments. Our findings were in line with earlier publications, that is, 2 recent studies also reported difficulties (55% and 50% baseline and follow-up data, respectively [43,45]) in collecting physical activity data using a triaxial pedometer or accelerometer on the hip. A possible explanation for these missing values might be the instruction to wear the monitor during waking hours compared with a more compliant 24-hour protocol [55]. In addition, one has to consider the trade-off between the study power and validity of the collected physical activity data [55]. However, missing follow-up data were mainly because of early revascularization or hospitalization (3 participants) and lack of valid combinations of at least three weekdays, Saturday, and Sunday (4 participants). Thus, missing follow-up data in our pilot study were considered to be the result of the selected analysis protocol [40] and patient hospitalization at follow-up.

### Limitations

Further limitations include the generalizability of this pilot intervention, which was part of developing a larger trial and should be interpreted as such. Only one researcher provided feedback and evaluated all outcomes. With regard to monitoring and feedback, calls or emails were structured to discuss walking training, elastic band training, and progression toward the new week. Although we incorporated some behavioral change techniques through the addition of sports watch technology (eg, self-monitoring), we did not assess and evaluate the underlying psychosocial constructs or the distinct effect of each behavioral change technique on effectiveness [31]. However, our evaluation of satisfaction and acceptance of technology could drive future research to evaluate and design technology to support long-term behavioral changes in a home-based environment. We did not assess the similarity between the uploaded exercise sessions and the actual walking prescription, which limits the interpretation of quantity and quality of exercise prescription [19]. One barrier to this approach was the presence of uninterpretable GPS signals (eg, because of a lack of satellite connections or an obstructed environment [high buildings or trees]) [46]. Similarly, although technology was well accepted, patients often reported the need for technical assistance during setup and interpretation [56]. In addition, it is well known that self-reported adherence rates from walking sessions and resistance training might result in overreporting [19]. However, our pilot did show good adherence to the walking sessions in comparison with other physiotherapy-led home-based exercise programs (67%) [19]. Moreover, our sample was generally fit in terms of activities of daily life measured by the SPPB and TUG total scores, which resulted in a ceiling effect [57]. Although both SPPB and TUG possess prognostic (eg, mortality [58]) information in patients with IC, high baseline scores impose an important risk for type II errors in clinical trials [57]. Therefore, physical fitness levels can be overestimated, as can be seen from the comparison of our measured time data with normative values [57]. As such, future studies are encouraged to report the measured time for both chair-stand and 4-m gait speed tests [57].

### Conclusions

This observational pilot study has shown that patients with IC are satisfied and accept technology to monitor and guide a home-based combined exercise program through remote feedback. Participants did not prefer resistance training over walking exercise; however, a general positivity toward the combined intervention was reflected in clinically relevant improvements in subjectively reported walking distances and quality of life.

## Appendix

Multimedia Appendix 1 Elastic band resistance exercises: instructional video.

Multimedia Appendix 2 Tables and figures with results on primary and secondary outcomes.

Multimedia Appendix 3 CONSORT-eHEALTH checklist (V 1.6.1).

## Abbreviations

ABI: Ankle-Brachial Index
ESES: Exercise Self-Efficacy Scale
HBET: home-based exercise therapy
HONOR: Home-Based Monitored Exercise for the PAD
IC: intermittent claudication
LEAD: lower extremity artery disease
MET: metabolic equivalent
MWD: maximal walking distance
PFWD: pain-free walking distance
SET: supervised exercise training
SPPB: Short Physical Performance Battery
TSK: Tampa Scale of Kinesiophobia
TUG: Timed Up and Go
WIQ: Walking Impairment Questionnaire
