# Supplementary material for: Satisfaction and Acceptability of Telemonitored Home-Based Exercise in Patients With Intermittent Claudication: Pragmatic Observational Pilot Study
Source: JMIR Rehabil Assist Technol. 2021 Mar 22;8(1):e18739. doi: 10.2196/18739 (PMC8108570; doi:10.2196/18739)
Supplement: Multimedia Appendix 2 [file rehab_v8i1e18739_app2.docx]

# Multimedia appendix 1

# Table S1

#### Participant information

| Characteristics of the studied sample (n=20) at baseline | | | | | | | | | | | | | | | | | |
| --- | --- | --- | --- | --- | --- | --- | --- | --- | --- | --- | --- | --- | --- | --- | --- | --- | --- |
| *Demographics* | | | *Cardiovascular Risk* | | | | | *Symptoms* | | | | *Walking distance and questionnaires* | | | | | |
| Participants | Gender | Age | BMI | DM | DYSL | HTN | SMO | RF | LOC | TIME | ABI | PFWD | MWD | VascuQOL | TSK | WIQ | ESES |
| *P1* | **♀** | 64 | 21.2 |  | ● | ● | ● | 3 | LC | 7.5 | 0.75 | 598 | 828 | 5.52 | 30 | 0.55 | 32 |
| *P2* | **♀** | 75 | 29.6 |  | ● | ● |  | 1 | RC | 2 | 0.70 | 126 | - | 5.96 | 31 | 0.62 | 34 |
| *P3* | **♀** | 58 | 27.8 |  | ● |  | ● | 3 | LC | 48 | 0.60 | 73 | 561 | 5.52 | 39 | 0.72 | 35 |
| *P4* | **♂** | 62 | 34.1 | ● | ● | ● | ◌ | 3 | BH | 30 | 0.35 | 123 | 306 | 3.96 | 41 | 0.32 | 30 |
| *P5* | **♂** | 69 | 21.7 | ● | ● | ● | ◌ | 1 | LC | 18 | 0.70 | -* | *-** | 5.56 | 37 | 0.87 | 36 |
| *P6* | **♂** | 71 | 19.4 |  | ● | ● | ◌ | 3 | BC | 120 | 0.45 | 158 | 300 | 4.64 | 47 | 0.59 | 38 |
| *P7* | **♂** | 50 | 29.3 |  | ● |  | ◌ | 3 | BCH | 15 | 0.45 | 138 | 227 | 4.52 | 38 | 0.78 | 33 |
| *P8* | **♂** | 66 | 36.2 |  | ● |  | ◌ | 3 | BC | 36 | 0.60 | 343 | 448 | 4.36 | 45 | 0.33 | 29 |
| *P9* | **♂** | 71 | 29 | ● | ● | ● | ◌ | 3 | BC | 228 | 0.80 | 95 | 141 | 5.32 | 43 | 0.40 | 38 |
| *P10* | **♂** | 79 | 26.8 |  | ● | ● | ◌ | 3 | BC | 12 | 0.85 | 84 | 142 | 6.20 | 29 | 0.58 | 37 |
| *P11* | **♂** | 69 | 31.5 |  | ● | ● | ◌ | 2 | RC | 22 | - | 284 | 414 | 5.36 | 34 | 0.64 | 40 |
| *P12* | **♂** | 67 | 30.5 | ● | ● | ● | ◌ | 2 | LC | 12 | 0.70 | 196 | 611 | 5.76 | 27 | 0.79 | 37 |
| *P13* | **♂** | 71 | 26.4 |  | ● | ● | ◌ | 2 | RC | 6 | 0.55 | 334 | 559 | 5.32 | 35 | 0.70 | 32 |
| *P14* | **♂** | 77 | 30.6 |  | ● | ● | ◌ | 2 | BC | 90 | 0.60 | 210 | 507 | 4.96 | 38 | 0.44 | 37 |
| *P15* | **♀** | 50 | 34.8 | ● | ● | ● | ◌ | 2 | BC | 11 | 0.70 | 226 | 322 | 3.32 | 41 | 0.68 | 32 |
| *P16* | **♂** | 56 | 29.7 |  | ● | ● | ◌ | 1 | BC | 7.5 | 0.60 | 142 | 347 | 6.16 | 32 | 0.58 | 38 |
| *P17* | **♂** | 61 | 32.7 |  | ● | ● | ◌ | 1 | BC | 12 | 0.85 | 562 | 600 | 5.44 | 38 | 0.58 | 40 |
| *P18* | **♀** | 41 | 27.2 |  | ● |  | ● | 3 | BT | 36 | 0.60 | 97 | 441 | 3.36 | 40 | 0.44 | 33 |
| *P19* | **♂** | 54 | 23.9 |  | ● |  |  | 3 | RFC | 21 | 0.30 | 165 | 365 | 5.28 | 29 | 0.63 | 35 |
| *P20* | **♂** | 81 | 25.1 |  | ● |  |  | 2 | RC | 6 | 1.15 | 183 | *-* | 6.28 | 39 | 0.83 | 39 |
| Overall | 15♂ 5♀ | 64.6 | 29.0 | 25% | 100% | 70% | 15% | - | 18C | 37 | 0.65 | 218 | 418 | 5.14 | 36.7 | 0.60 | 35.3 |
| *Abbreviations: Cardiovascular risk factors****;*** *BMI = body mass index (kg/m²). DM = diabetes mellitus. DYS = dyslipidemia. HTN = hypertension. SMO = smoking (◌ ex-smoker. ● current smoker (including e-cigarette)); RF= Rutherford- class. LOC = location of symptoms (L = left. R = right and B = bilateral; F = foot. C = calf. T= thigh and H = hip). TIME = duration of symptoms (in months). ABI = ankle-brachial index (Doppler data most affected limb); PFWD = pain-free walking distance (meter). MWD = maximal walking distance (meter). TSK = Tampa scale for kinesiophobia. WIQ = Walking impairment questionnaire and ESES = Exercise self-efficacy scale. Overall data are either counts (n. %) or mean values. P5*: did not experience any IC symptoms during baseline and follow-up treadmill testing although anamnesis reported complaints were typical of IC.* | | | | | | | | | | | | | | | | | |

# Table S2

### Questionnaires

|  | *Baseline* | *Follow-up* | *Difference* | *P-value* |
| --- | --- | --- | --- | --- |
|  |  |  |  |  |
| *VascuQOL* | n=20 | n=20 |  |  |
| Pain | 4.76 (0.86) | 5.18 (0.74) | +0.41 (0.84) | *0.040* |
| Symptoms | 4.84 (1.17) | 4.99 (1.18) | +0.15 (0.63) | 0.301 |
| Activities | 5.10 (0.81) | 5.64 (0.87) | +0.54 (0.59) | *<0.001* |
| Emotions | 5.43 (IQR: 1.00) | 6.14 (IQR: 1.29) | +0.36 (IQR: 0.86) | 0.064 |
| Social* | 6.50 (IQR: 2.00) | 7.00 (IQR: 2.50) | 0.00 (IQR: 0.50) | 0.556 |
| *Total* | 5.14 (0.87) | 5.50 (0.91) | +0.36 (0.55) | *0.009* |
| *WIQ* | n=20 | n=20 |  |  |
| Distance | 0.45 (0.24) | 0.63 (0.27) | +0.18 (0.29) | *0.013* |
| Speed | 0.60 (0.21) | 0.63 (0.21) | +0.03 (0.21) | 0.528 |
| Stairs | 0.77 (IQR: 0.27) | 0.80 (IQR: 0.32) | +0.03 (IQR: 0.14) | 0.552 |
| Total | 0.60 (0.16) | 0.68 (0.19) | +0.08 (0.17) | 0.056 |
| *TSK* | n=20 | n=20 |  |  |
| Total* | 38.0 (IQR: 8.50) | 35.5 (IQR: 10.25) | -1.0 (IQR: 4.25) | 0.169 |
| *ESES* | n=20 | n=20 |  |  |
| Total | 35.50 (IQR: 5.25) | 37.50 (IQR: 5.50) | +1.00 (IQR: 2.50) | 0.178 |

**Supplementary table 2.** Data mean (SD) or median (IQR)*. Non-parametric testing (Wilcoxon-Signed Rank) is performed when the difference scores are not normally distributed (*)

# Table S3

#### Physical fitness & physical activity

|  | *Baseline* | | *Follow-up* | *Difference* | *P-value* |
| --- | --- | --- | --- | --- | --- |
|  |  | |  |  |  |
| *SPPB* | n=20 | | n=20 |  |  |
| Balance* | 4 (IQR: 1) | | 4 (IQR: 0) | 0 (IQR: 1) | 0.066 |
| 4-m gait speed * | 4 (IQR: 0) | | 4 (IQR: 0) | 0 (IQR: 0) | 1.000 |
| 4-m gait speed (sec) | 4.14 (0.54) | | 4.02 (0.61) | -0.11 (0.44) | 0.263 |
| chair stand* (n=19) | 3 (IQR: 1) | | 4 (IQR: 1) | 0 (IQR: 1) | 0.565 |
| chair stand (sec) (n=19) | 11.33 (2.50) | | 10.93 (2.14) | -0.40 (1.55) | 0.275 |
| *Total** (n=19) | 11 (IQR: 2) | | 11 (IQR: 1) | 0 (IQR: 1) | 0.060 |
| *TUG* | n=20 | | n=20 |  |  |
| TUG (t) | 8.26 (1.32) | | 8.06 (1.35) | -0.20 (0.86) | 0.305 |
| *Physical activity* | n=19 | n=11 | n=11 | n=11 |  |
| Light PA (min/day) | 185  (IQR: 72) | 185  (IQR: 89) | 209  (IQR: 127) | 5.0 (64) | 0.800 |
| Moderate PA (min/day) | 59  (IQR: 59) | 64  (IQR: 69) | 67  (IQR: 48) | -13.8 (42) | 0.299 |
| Vigorous PA (min/day)* | 2.0  (IQR: 4.3) | 2.1  (IQR: 4.3) | 1.3  (IQR: 15) | -0.0  (IQR: 2.8) | 0.673 |
| Steps (number/day) | 5297  (IQR: 3118) | 4507  (IQR: 3876) | 4537  (IQR: 1736) | -443 (1452) | 0.335 |
| Sedentary time (min/day) | 1181  (IQR: 130) | 1181  (IQR: 164) | 1147  (IQR: 128) | -0.9 (92) | 0.975 |

**Supplementary table 3**. Data mean (SD) or median (IQR) based on distribution. Non-parametric testing (Wilcoxon-Signed Rank) is performed when the difference scores are not normally distributed or data is ordinal scaled (*).

# Figure S1

#### Walking distance

#### Pain-free walking distance & maximal walking distance


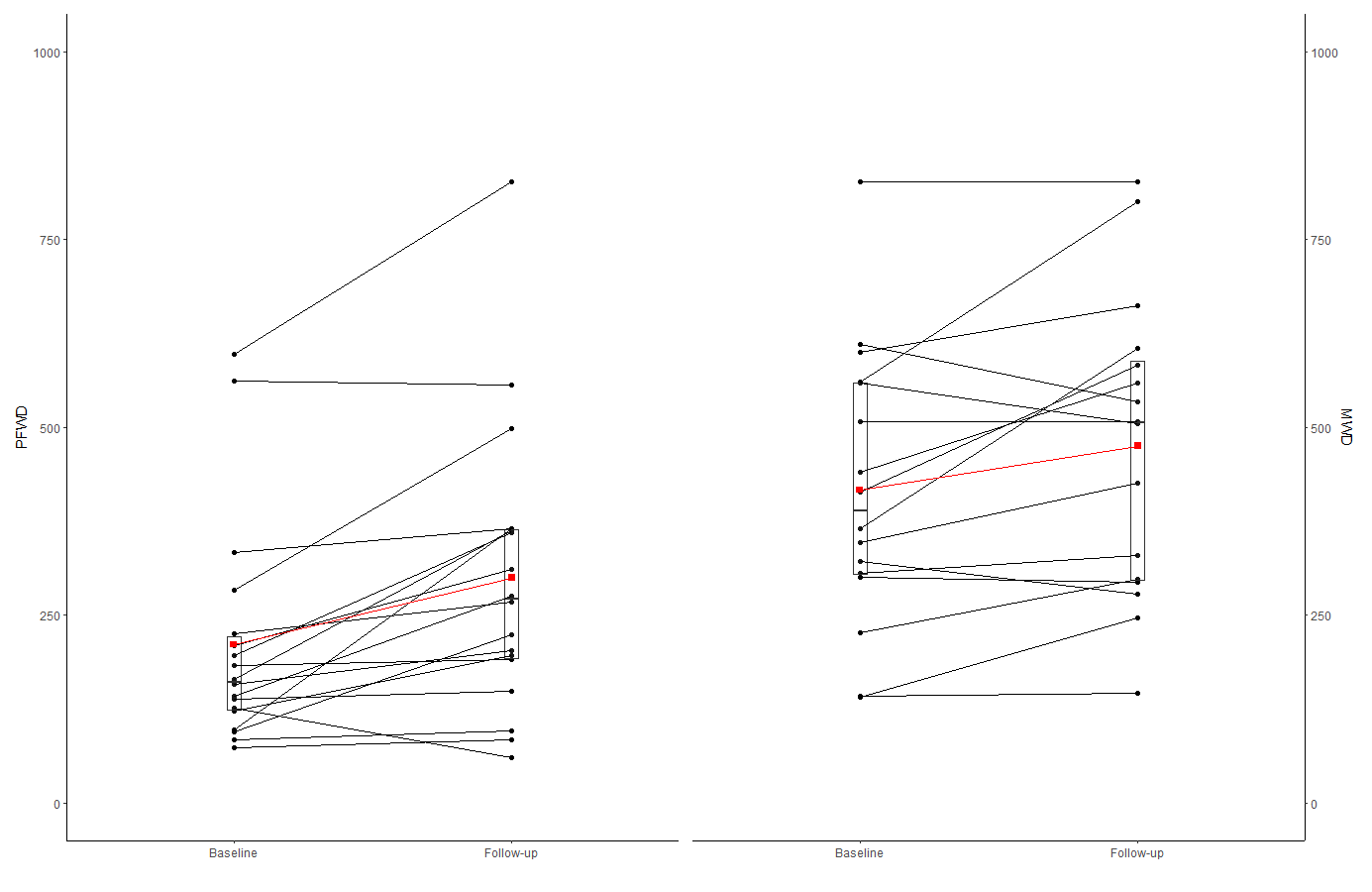


**Supplementary figure 1.** Pain-free (n=18; left side) and maximal walking distances (n=16; right side) displayed as connected scatters at baseline and follow-up.

Boxplots are overlapping and mean scores are indicated red.

# Table S4

#### Sensewear: Physical Activity

| *Sensewear PA* | *95% criterion* | *90% criterion* |
| --- | --- | --- |
| *Baseline* | | |
| Weekdays (>= 3 days) | 18 (90%) | 19 (95%) |
| Weekend (>= 2 days) | 15 (75%) | 19 (95%) |
| Total | 15 (75%) | 19 (95%) |
|  | | |
| Follow-up | | |
| Weekdays (>= 3 days) | 14 (70%) | 14 (70%) |
| Weekend (>= 2 days) | 9 (45%) | 12 (60%) |
| Total | 8 (40%) | 11 (55%) |

**Supplementary table 4.** Analysis of PA data using different wear-time (95% and 90%) cut-offs.

# Figure S3

### Physical activity data analysis

# Figure S4

#### Resistance exercises

| **Plantar flexion**  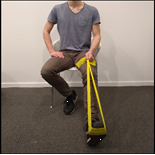 | **Hip flexion**  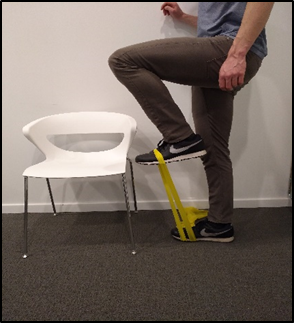 |
| --- | --- |
| **Hip extension**  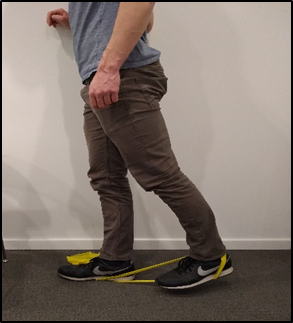 | **Hip abduction**  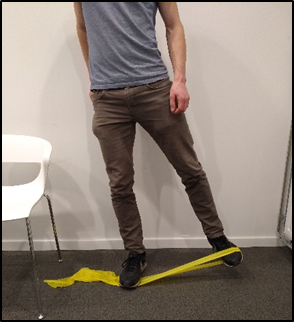 |
